# Videocapsule retention: role of surgical treatment (a case report)

**DOI:** 10.11604/pamj.2015.22.64.4300

**Published:** 2015-09-23

**Authors:** Rachid Boufettal, Yassine Fahmi, Saad Rifki Jai, Farid Chehab

**Affiliations:** 1 General Surgery Department III, UHC Ibn Rochd, Casablanca, Morocco

**Keywords:** Video capsule, retention, small bowel stenosis, surgical extraction, stricturoplasty

## Abstract

Video capsule endoscopy (VCE) is a safe innovative tool for investigating obscure gastrointestinal diseases. The capsule is usually excreted with faeces within 24-48 h. Retention of capsule rarely occurs, and it usually depends on the indication of VCE. Retention may long remain asymptomatic or manifest as subocclusif syndrome. Acute complications of retention are very rare but can be life-threatening illness. Surgical approach is considered effective to retrieve the retained capsule, treat the pathology responsible and prevent acute complications. We report the case of a 30 years old patient, followed for Crohn's disease. She received during the assessment reviewed by VCE that has been held for three months. The retention caused subocclusif symptoms of which had needed surgically procedure. Treatment consisted of remove the VCE and repair of stenosis of small bowel by stricturoplasty.

## Introduction

Videocapsule endoscopy (VCE) has become a routine investigative tool in the diagnosis of small bowel diseases. Retention of the VCE is a serious complication of this examination. The retention leads to the occurrence of acute intestinal obstruction or intestinal perforation requiring emergency surgery [1, 2]. We report the case of VCE retention on small bowel stenosis due to CROHN disease.

## Patient and observation

**Figure 1:**
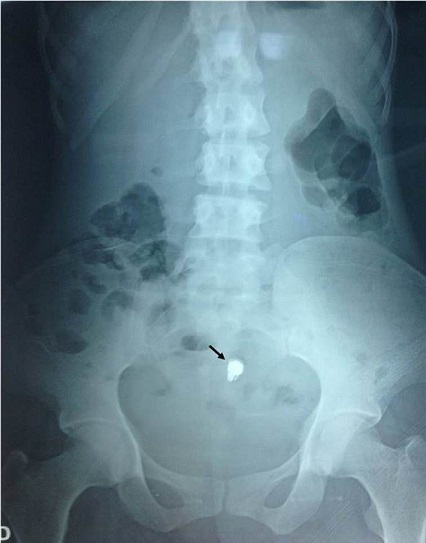
Plain abdominal radiography showing retained videocapsule (arrow)

**Figure 2:**
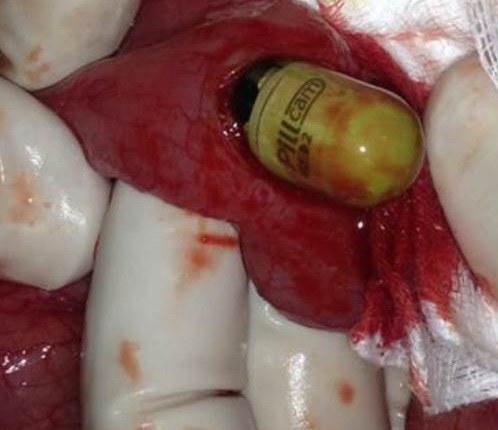
Photo during surgery showing video capsule extraction by enterotomy

**Figure 3:**
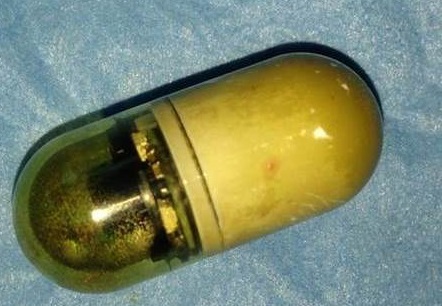
Video capsule after extraction

A 30-year-old woman without medical history had undergoing an exploration by VCE three months ago for digestive symptoms as diffuse abdominal pain, chronic constipation, Iron-deficiency anemia and alteration of general state. The patient has not eliminated the capsule after 48 hours. Plain abdominal radiography showed persistence of VCE (Figure 1). Thereafter, the patient began to exhibit repeated subocclusif syndrome. She underwent surgical exploration that showed wall thickening and incomplete strictures at multiple levels of small bowel compatible with ileal Crohn′s disease. The VCE was enclosed in stricture located 2 meters from duodenojejunal junction. A longitudinal enterotomy on the stricture with VCE extraction was performed (Figure 2, Figure 3). The enterotomy was repaired by stricturoplasty. The postoperative course was uneventful.

## Discussion

Capsule retention is defined by the International Conference on Capsule Endoscopy (ICCE) 2005 as having a capsule endoscope remain in the digestive tract for minimum two weeks. Capsule retention is further defined as the capsule remaining in the bowel lumen unless it is recovered medically, endoscopically or surgically [3].

Retention rate of VCE depends mainly on its indications. It goes from 0% in subjects without any gastrointestinal disease to 21% in cases of intestinal obstruction [4]. This rate is 5% in patients suspected Crohn's disease. Liao et al [5], reported 184 cases of retention (1,4%) among 22840 explorations with VCE of which 2,6% occurred on Crohn's disease, 1,2% on obscure gastrointestinal bleeding and 2,1% on gastrointestinal neoplasm. Furthermore, AINS induced enteropathy, post-operativestenosis, ischemia and radiation enteritis can occur retention [5, 6]. Meckel's diverticulum, peptic ulcer, cryptogenic multifocal stenosing enteritis can rarely be involved with frequencies of less than 2% of total capsule retention. In our patient, there tention occurred probably on crohn's disease [4].

Retention of capsule is mostly asymptomatic. Furthermore, it causes subocclusif symptoms [2, 5]. Retention can contribute to etiological diagnosis and the level of obstruction [2]. Some acute complications due to retention of VCE were reported in literature. In a study of 2300 explorations by VCE, six cases have acute intestinal obstruction [1]. One case of intestinal perforation 2 months after par VCE examination for exploration of anemia was reported [7].

The management of capsule retention can undergo expectant, medical and endoscopic procedure or even surgical intervention. Medical treatment is based on anti-inflammatories, colonic preparation or rectal enema [1, 8]. Surgery allows not only the extraction of the capsule but can also contribute to treatment of the etiology of retention and prevents its complications [5, 7]. The longest duration of retention is reported by Bhattarai et al [9], the capsule was retained on ileorectal anastomosis without complication during 4,5 years and it was successfully removed by endoscopic procedure. Hauser et al [10] reported two cases of VCE retention. In the first case, VCE was located in proximal jejunum and successfully removed by enteroscopy, in the second case; VCE was located in rectum and removed by surgery after failure colonoscopy. Liao et al [5] reported spontaneous elimination or by medical treatment in 15%, by endoscopic procedure in 12% andby surgery in 58,7% of the cases with retention capsule. One death attributed to surgery complication was reported [1]. Our patient underwent surgery extraction with treatment of stenosis by stricturoplasty. The postoperative course was uneventful.

## Conclusion

VCE retention is a serious complication this examination. Although rare, we must keep in mind the possibility of an acute complication of retention of the VCE. Surgical treatment must be considered in case of failure of endoscopic extraction and if a cause of retention requires surgery. Surgical treatment allows the extraction of the capsule, the treatment of the cause and prevents acute complications of retention.
